# Evaluation of MR Spectroscopy and Diffusion-Weighted MRI in Postmenopausal Bone Strength

**DOI:** 10.7759/cureus.327

**Published:** 2015-09-21

**Authors:** Kanhaiya Agrawal, Yatish Agarwal, Rajesh K Chopra, Achla Batra, Ranjan Chandra, Brij B Thukral

**Affiliations:** 1 The Department of Diagnostic Radiology and Imaging, Safdarjung Hospital; 2 Central Institute of Orthopedics, Safdarjung Hospital; 3 The Department of Obstetrics & Gynaecology, Safdarjung Hospital

**Keywords:** osteoporosis, diffusion weighted imaging, bone marrow, spectroscopy, bone mineral density

## Abstract

Aim: To prospectively investigate the role of MR spectroscopy (MRS) and diffusion-weighted magnetic resonance imaging (DWI) in assessing vertebral marrow changes in postmenopausal women.

Materials and Methods: Fifty postmenopausal women, who underwent dual-energy x-ray absorptiometry of the spine, were divided into three bone density groups (normal, osteopenia, and osteoporosis) based on T-score. Both MRS and DWI of the L3 vertebral body were performed to calculate the marrow fat content and apparent diffusion coefficient (ADC). The results were compared between three groups and correlated with BMD.

Results: Vertebral marrow fat content was significantly increased in the osteoporotic group when compared with that of the osteopenic group and the normal bone density group. ADC values in the osteoporotic, osteopenic, and normal bone density groups were 338, 408 and 464, respectively, with statistically significant differences (P < 0.001). A statistically significant positive correlation between T-scores and ADC existed (r=0.694, p value <0.001). The vertebral marrow fat content was negatively correlated to the bone density (r=–0.455, p< 0.001) and to marrow ADC (r= -0.302, p < 0.05).

Conclusion: The postmenopausal women with osteoporosis exhibited a corresponding increase in vertebral marrow fat content as the bone density decreased. Marrow fat content and ADC were related to the bone density. MRS and DWI are helpful in evaluating the bone marrow changes in postmenopausal women.

## Introduction

Osteoporosis is a systemic disorder characterized by a reduction in bone mass and degeneration of microstructure of bone tissue, resulting in increased bone fragility and fracture. It is the most common metabolic bone disorder, affecting one in four women and one in eight men older than 50 years [1]. 

The physiologic homeostatic processes affect the dynamic constituents of the bone, including the fat quotient in the marrow, the ratio of red to yellow marrow, and the architecture of the trabeculae. Currently, osteoporosis is diagnosed primarily by determination of bone mineral density (BMD) [3]. As a measure of the amount of matter per cubic centimeter of bones, the clinical utility of BMD lies as an indirect determinant of osteopenia and osteoporosis, which, in turn, measures the fracture risk. Several studies have demonstrated that with ageing and bone weakening, the marrow fat content progressively increases. Additional fat cells replace age-related trabecular bone loss and fill bone resorption cavities [3-4]. This increased marrow fat content could therefore be taken as the indicator of bone loss [5]. Magnetic resonance imaging (MRI) may non-invasively provide an insight into the pathogenesis of osteoporosis in three aspects—marrow fat content, diffusion, and perfusion. The recent developments in the field of magnetic resonance (MR) imaging – MR spectroscopy and diffusion weighted MRI – have opened new vistas of fashioning an entirely novel non-invasive, radiation-free technique, which could substitute or supplement DEXA in evaluation of bone strength both in qualitative and quantitative terms. Proton MR spectroscopy can be a useful tool for quantitative evaluation of bone tissue components, since it is capable of separating the total bulk MR signal into its components of distinct lipid and water signals [3-4]. Diffusion-weighted MR imaging measures the random, translational motion of water molecules in a biologic tissue, which reﬂects its microstructure and the tissue-speciﬁc diffusion capacity [6-7]. The aim of the present study was to investigate the potential of magnetic resonance spectroscopy (MRS) and diffusion-weighted MRI (DWI) in assessing bone marrow changes in postmenopausal women.

## Materials and methods

Fifty postmenopausal women were recruited randomly from patients who underwent dual-energy x-ray absorptiometry (Osteocore 3, Platinum) of the spine over a period of 18 months between November 2012 and April 2014. Prospective MRI was performed in all 50 patients. Patients suffering from a known pre-existing bone disease, such as tumour, metastasis, or obvious osteoarthritis, or receiving a drug therapy that may affect BMD were excluded from the study. Informed consent from all participants and prior ethical approval for the study were obtained from the Office of the Medical Superintendent, VMMC and Safdarjung Hospital, New Delhi, according to institutionally approved procedures and regulations. The bone density of the vertebral body was expressed as a T-value at L3 measured by posteroanterior projection. According to the World Health Organization criteria, the participants were categorized into three groups based on the DEXA results:

(1) normal BMD (T<-1)

(2) osteopaenia (-2.5<T<-1)

(3) osteoporosis (T<-2.5) [8].

MRI was performed using a 1.5T Philips Achieva MRI scanner installed in the hospital. A spinal array surface coil was used for signal acquisition. Tl-weighted and T2-weighted imaging of the lumbar spine in the sagittal plane were acquired by using a fast spin-echo sequence. These images were used to identify pre-existing abnormalities of the lumbar vertebrae and to guide the position of a volume of interest within the boundaries of the L3 vertebral body for DWI and MRS. After local shimming and gradient adjustments were performed, MRS was obtained by using a point-resolved MR spectroscopic sequence (TR 5000 ms; TE25 ms) at a spectral bandwidth of 1000, and setting water suppression voltages to zero. A volume of interest with dimensions 1 (length) x 1 (width) x 1 (height) cm3 was placed centrally in the L3 vertebral body. Peak amplitudes of the water resonance and the main lipid peak were measured. The bone marrow fat fraction (FF) was calculated mathematically by using the following formulae:

First, the lipid-water ratio (LWR) was derived as follows:

     Lipid-water ratio (LWR) =  Lipid peak amplitude/Water peak amplitude

Subsequently, the bone marrow fat fraction (FF) derived:

     Fat fraction (FF) =  Lipid-water ratio (LWR)/(LWR+1)

Sagittal lumbar vertebral DWI images were acquired by using a spin-echo single-shot echo planar imaging sequence (TR 1000 ms; TE 70 ms; section thickness 5 mm; field of view 28 mm; and matrix 128 x 256). Two diffusion-weighted images were obtained with diffusion sensitivity parameters (b-values) of 0 and 1000 s/mm2 in three planes (x, y, z). The isotropic DWI images with b values around 1000 s/mm2 produced good resolution and high signal-to-noise ratio (SNR) [9]. ADC values were calculated from the automatically generated ADC maps of the DW images. Data are presented as a mean ± standard deviation. Variables were compared among the three bone density groups with the one-way analysis of the variance test. Pearson correlation coefficients were calculated to assess the linear relationship between pairs of variables. P < 0.05 was considered statistically significant.

## Results

A total of 50 participants, aged 40-73 (52.46) years old, underwent both DEXA and conventional MRI with DWI and MRS. The number of participants in the osteoporotic, osteopaenic, and normal BMD groups was 15, 18, and 17, respectively.

Vertebral marrow fat content increased as bone density decreased. The mean values of FF were the greatest in the osteoporotic (50 ± 11%) subgroup, a little less in the osteopenic subgroup (42 ± 10%), and the least in the normal subgroup (31 ± 14%). The bone marrow ADC (x10^-6^mm^2^/s) was significantly decreased in the osteoporotic group (464 ± 60) and the osteopenic group (408 ± 68) in relation to the normal group (338 ± 70), respectively (Table 1).

**Table 1:** Comparative Mean Values of FF and ADC

Category of Subjects	Mean Fat Fraction ± SD	Mean ADC ± SD
Normal	0.31 ± 0.14	464.94 ± 60.17
Osteopenic	0.42 ± 0.10	408.06 ± 68.28
Osteoporotic	0.50 ± 0.11	338.13 ± 70.86

Data was analyzed by using the Pearson correlation coefficient to consider the linear relationship between the T–score and the fat fraction. A significant negative correlation was observed between the two: r = –0.450 and p < 0.003. Similarly, the linear relationship between the BMD and the fat fraction was also assessed. A significant negative correlation was found between the BMD and vertebral marrow fat fraction (r = –0.3455 and p < 0.001).

Linear relationship between the T-score and ADC were also determined by using bivariate correlation and computing the Pearson correlation coefficient (r). A significant positive correlation was observed between the BMD and bone marrow ADC with r = 0.693 and p < 0.0001. Linear relationship between the two variables, BMD and ADC, in all 50 subjects was determined by using the bivariate correlation and computing the Pearson correlation coefficient (r). A significant positive correlation was observed between the BMD and bone marrow ADC with r = 0.621 and p-value < 0.0001.

The linear relationship between the two variables, FF and ADC, in all 50 subjects was determined by using the bivariate correlation and computing the Pearson correlation coefficient (r). A significant negative correlation was observed between the FF and the bone marrow ADC with r = -303 and p-value < 0.05.

## Discussion

A common osseous condition characterized by low bone mineral density and ensuing fragility fractures, osteoporosis has recently gained excited considerable interest in the medical fraternity due to a variety of reasons: One, it has become a major healthcare problem worldwide because of the growing elderly population [11]. Some recent studies have proposed that increased bone marrow fat could have an etiologic link to reduced bone density and that measures to inhib­it marrow adipogenesis could have a positive effect on bone strength [11]. Increasing vertebral marrow fat content may cause errors in DEXA measurements with misrepresentation of the patient's true bone status when BMD results are interpreted in terms of the T-scores [8, 12].

Based on this fundamental in vitro data, techniques for non-invasive measurement of bone marrow fat have been searched for. Two such techniques based on MRI, proton MR spectroscopy and diffusion-weighted MRI, have come under scrutiny for their potential diagnostic value in the recent years.

Set in this background, this study was primarily done to determine if the newfound parameter – FF and ADC of bone marrow obtained on spectroscopy and diffusion weighted imaging, respectively – could stand up as a reliable measure of bone strength. Further, it sought to pronounce a preliminary finding on its diagnostic significance as a measure of osseous strength in comparison to the commonly used bone signature, bone mineral density, and if it could be used to supplant DEXA, a technique that carries the risks associated with ionizing radiation.

The methodology used was simple. DEXA was used to measure the bone mineral density (BMD), and on the basis of the T–score, the 50 subjects were categorized into three subgroups: normal 17 (34%), osteopenic 18 (36%), and osteoporotic 15 (30%). Significant differences were observed in the bone mineral density among these three subgroups: normal subjects had a BMD between 0.94 to 1.19 g/cm^2^ and osteopenic subjects between 0.74 to 0.99 g/cm^2^, whereas osteoporotic subjects had a BMD between 0.44 to 0.84 g/cm^2^. The calculated p-value for the three subgroups was found to be significant. These results compare well with the established literature.

In each subject, proton MR spectroscopy was carried out to determine the lipid-water ratio and calculate the bone marrow fat fraction with L3 vertebra as the reference. Studies propose that the L3 vertebra is the least likely to be affected by the age-related degenerative changes, a change found commonly in the lower lumbar vertebra [5].

Results demonstrate a significant difference in the fat fraction (FF) of the three subgroups. The mean values of FF were greatest in the osteoporotic subgroup (0.50), a little less in the osteopenic subgroup (0.42), and the least in the normal subgroup (0.31). This data confirmed an inverse relationship between vertebral bone marrow fat and bone mineral density (r=–0.455, p< 0.001) and also, with the T-scores (–0.450 and p <0.003). These results match closely with at least two other recent studies [7-8]. When considered singly, the diagnostic significance of the fat fraction (FF) in being able to distinguish the spectrum of normal, osteopenic, and osteoporotic bone strength was found comparable to BMD, with a calculated p-value of < 0.001.

In each subject, DW-MRI was carried out to determine the ADC value of bone marrow with L3 vertebra as the reference. Results demonstrate a significant difference in the obtained ADC value of the three subgroups. The mean values of ADC(x10-6mm2/s) were lowest in osteoporotic subgroup 338, little more in osteopenic subgroup 408, and greatest in normal subgroup 464 and a statistically significant difference (p<0.001) was observed between the mean ADC values of any two groups. The obtained data confirmed a positive linear relationship between vertebral bone marrow ADC and bone mineral density r=0.621, p-value <0.001 and also, with T-score, r=0.694, p-value <0.001. These results also matched closely with other recent studies [10-15]. We also observed that the diagnostic significance of bone marrow ADC in being able to distinguish the spectrum of normal, osteopenic and osteoporotic bone strength was found comparable to BMD, both having a calculated p-value of <0.001.

The basis for this direct relationship between bone marrow fat fraction, ADC, and bone mineral density, however, needs to be elucidated. Based on histomorphometric studies and MR spectroscopic studies, the connecting link between these two entities appears to be the ‘bone marrow fat’. Histomorphometric studies have demonstrated increased bone marrow fat associated with osteoporosis [13-24].

It has also been demonstrated in several studies using histomorphometry and MR spectroscopy that increased amounts of fat in the tissues leads to decrease in the diffusion capacity and, hence, decreased apparent diffusion coefficient values [7, 25-31]. 

Thus, ADC value reflects the marrow fat which in turn is closely related to BMD. This may be the underlying reason behind the low ADC in the individuals with low BMD as obtained in a few recent studies, including our study.

The present study demonstrates the significance of bone marrow FF and ADC values in being able to identify between normal, osteopenic, and osteoporotic bone strength. Results obtained indicate that bone marrow FF and ADC value can identify bone weakening in equal measure as BMD (p<0.001) can. Thus, its diagnostic strength to detect bone weakening stands validated. 

In essence, on the basis of the results of this study, it can safely be proposed that the use of MR spectroscopy and diffusion-weighted MRI for measurement of weakening is liable to evolve as an authoritative extension of diagnostic MR imaging in the select domain of osteoporosis therapeutics. However, before applying these results to a larger population, the findings need further corroboration in larger series.

## Conclusions

With the increase in age, the vertebral marrow fat content increases and the bone density decreases. This increase in marrow fat content, or fat fraction, can be quantitatively determined with magnetic resonance spectroscopy; the diffusion capacity and the bone marrow ADC obtained on diffusion-weighted MRI exhibits a corresponding decrease.

Thus, MRI has the distinctive ability to quantify fat fraction and ADC as well as to distinguish the tissue characteristics of bone marrow in a non-invasive manner. Furthermore, the statistical significance of this virtue is comparable to that of bone mineral density assessed with DEXA, the hitherto gold standard. At the same time, diffusion-weighted MRI does not entail any radiation, a demerit that exists with DEXA.

This study can also serve as a reference for the bone marrow fat fraction and ADC values in the Indian population having normal as well as low bone mass, and could help differentiate other conditions affecting the bone marrow.
